# Synthesizing Rolling Bearing Fault Samples in New Conditions: A Framework Based on a Modified CGAN

**DOI:** 10.3390/s22145413

**Published:** 2022-07-20

**Authors:** Maryam Ahang, Masoud Jalayer, Ardeshir Shojaeinasab, Oluwaseyi Ogunfowora, Todd Charter, Homayoun Najjaran

**Affiliations:** 1 Department of Electrical and Computer Engineering, University of Victoria, Victoria, BC V8P 5C2, Canada; maryamahang@uvic.ca (M.A.); ardeshir@uvic.ca (A.S.); toddch@uvic.ca (T.C.); 2 Department of Mechanical Engineering, University of Victoria, Victoria, BC V8P 5C2, Canada; masoudjalayer@uvic.ca (M.J.); ogunfool@uvic.ca (O.O.)

**Keywords:** generative adversarial networks, fault detection and diagnosis, condition monitoring, signal processing, bearing fault detection

## Abstract

Bearings are vital components of rotating machines that are prone to unexpected faults. Therefore, bearing fault diagnosis and condition monitoring are essential for reducing operational costs and downtime in numerous industries. In various production conditions, bearings can be operated under a range of loads and speeds, which causes different vibration patterns associated with each fault type. Normal data are ample as systems usually work in desired conditions. On the other hand, fault data are rare, and in many conditions, there are no data recorded for the fault classes. Accessing fault data is crucial for developing data-driven fault diagnosis tools that can improve both the performance and safety of operations. To this end, a novel algorithm based on conditional generative adversarial networks (CGANs) was introduced. Trained on the normal and fault data on actual fault conditions, this algorithm generates fault data from normal data of target conditions. The proposed method was validated on a real-world bearing dataset, and fault data were generated for different conditions. Several state-of-the-art classifiers and visualization models were implemented to evaluate the quality of the synthesized data. The results demonstrate the efficacy of the proposed algorithm.

## 1. Introduction

Bearings are crucial parts of rotating machinery and are widely used in many industries; it was recorded that 44% of machine faults experienced in manufacturing industries are related to bearing failures [1]. Fault detection is a critical part of system design and maintenance because it helps to improve production efficiency resulting in reduced costs and accidents. Fault detection has gained interest in academia and industry, and has been a hot topic of research because of its significance [2]. Fault detection and diagnosis methods are generally classified into two groups: model-based methods and data-driven methods. In model-based methods, the model’s output and the actual system’s signals are used to generate several symptoms differentiating normal from faulty machine states; based on these symptoms, faults are determined through classification or inference methods. However, data-driven methods rely on the sensor data collected from the plant and usually use artificial intelligence (AI) to learn and classify characteristic fault features from data. AI plays an inevitable role in industry and manufacturing systems [3].

Deep learning methods, which are known for their capability to process massive amounts of data and are relatively robust against noise, are the best methods for intelligent fault detection [4]. Convolutional neural networks (CNNs) [5,6], stacked auto Encoders [7,8], and deep belief networks (DBNs) [9] are among the most studied algorithms that could reach very high accuracy. Industrial machines mainly operate in normal conditions, so there are more normal data than fault data, making the available data imbalanced. Even though some methods (such as one-class classification and novelty detection) can detect faults in such conditions, identifying the type of faults is not possible [10]. To solve this problem, generative algorithms can be employed to generate fault data. Generative algorithms are unsupervised learning paradigms that automatically discover patterns in input data so the model can produce new examples. Variational autoencoders (VAE) and generative adversarial networks (GANs) are the most famous generative models and have been wildly used for bearing fault detection [11,12], data augmentation [13], and predicting remaining useful life. By using generative algorithms, the problem of lack of samples and patterns in industrial data can be solved [14]. CGAN is a variation of GAN, which can generate conditional new data [15]. In [16], where datasets are limited and imbalanced, a conditional deep convolutional generative adversarial network is used for machine fault diagnosis. Yin et al. [17] also applied a data generation method based on the Wasserstein generative adversarial network and the convolutional neural network for bearing fault detection. These methods aim to extract the input data probability distribution and hidden information so that they can be sampled and used to generate new data. Moreover, the distribution of any condition is unique, so the distributions of unknown conditions can be found with the information of known ones and used to generate data for new conditions where fault data are not available.

**Figure 1:**
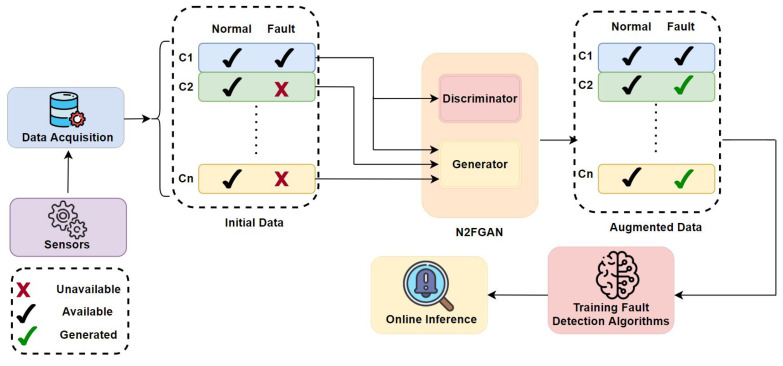
A flow diagram using N2FGAN for fault data generation.

In this paper, a novel method inspired by image-to-image translation [18] was introduced and tested on vibration signals to generate fault data from normal data. Pairs of normal and fault data were fed as inputs to the network at given conditions. After the training phase, the network can generate new fault data under different conditions. It has been assumed that no fault sample is available in other conditions, and the data generation is only conducted using normal data. The efficiency of the proposed method and the quality of generated data were evaluated using different classifiers and visualization methods. The paper’s organization is as follows: A literature review was conducted in Section 2. Next, Section 3 introduces some background theories and Section 4 elaborates on the proposed method and the normal to fault GAN (N2FGAN). The N2FGAN was tested on the Case Western Reserve University (CWRU) dataset and verified in Section 5, and finally, conclusions are available in Section 6. In Figure 1, a flow diagram using N2FGAN to generate synthetic fault data for new conditions in different systems is shown. $c_{1}$, $c_{2}$, …, $c_{n}$ refers to different working conditions.

## 2. Literature Review

Numerous AI techniques, such as traditional machine learning methods and deep learning approaches, have been used for fault recognition and diagnosis in roller ball bearings or rotating parts of machinery. Lei et al. [19] systematically reviewed the development of intelligent fault diagnosis (IFD) since the adoption of machine learning approaches, presenting the past, present, and future artificial intelligent approaches. Schwendemann et al. [20] surveyed machine learning in predictive maintenance and condition monitoring of bearings and studied different approaches to classify bearing faults and the severity detection.

Liu et al. [4] also presented a review of the literature on the applications of artificial intelligence algorithms for the fault detection of rotating machinery with a focus on traditional machine learning methods, such as the naive Bayes classifier, K-nearest neighbor (k-NN), support vector machine (SVM), and artificial neural network algorithms. In earlier years of intelligent fault diagnosis, traditional machine learning approaches involved collecting raw sensor data of various fault types, extracting features from the collected data, and developing diagnosis models from the features to automatically recognize the machine’s health status. Although traditional machine learning methods can automate the fault detection processes, these approaches cannot handle increasingly large data due to their low generalization performances, thereby reducing their accuracy in fault diagnosis. For instance, support vector machine classifiers can be applied to classification and regression problems. However, they do not perform well when applied to multi-class classifications or pairwise classification problems. Some approaches, such as SVMs, are computationally expensive and cannot deal with massive industrial data efficiently [21].

In recent times, deep learning paradigms for intelligent fault diagnoses have become prominent because they can automatically learn fault characteristics from the data without direct feature extraction. Moreover, they can handle large amounts of industrial data, which is one of the drawbacks of traditional machine learning methods; this has helped to reform intelligent fault diagnoses since the 2010s. Li et al. [22] reviewed the literature on the applications of deep learning methods for fault diagnoses, analyzing the deep learning approaches in relevant publications to point out the advantages, disadvantages, areas of imperfections, and directions for future research. Although the adoption of deep learning methods has led to many successes, these approaches assume that labeled data are sufficient for training diagnosis models [19]. However, this assumption is impractical, given the working conditions in most industries. The collected data are inadequate as machines seldom develop faults, and condition data healthier than faults are collected. So, even with deep learning approaches, the collected data are unbalanced and insufficient to train reliable fault diagnosis models. This poses some limitations in using intelligent fault diagnoses in industries. As mentioned earlier, the lack of fault data during the network training process is generally termed a small sample problem [23]. Researchers have come up with three significant ways to solve the small sample problem data augmentation-based, transfer learning/domain adaptation-based, and model-based strategies. The data augmentation and transfer learning-based methods attempt to increase the amount of data by generating similar data from the existing fault data; slightly modified copies of existing fault data are used to create synthetic data for training the neural network, transfer learning-based approaches use pre-trained networks from similar domains to train the new models in a bid to minimize the amount of data required for training.

GANs have unveiled promising capabilities in intelligent fault diagnoses for data argumentation and adversarial training purposes. They can be considered as potential solutions to the small sample problem because GANs can be used to generate additional data with the same distributions as the original data. The generative adversarial network was first introduced by Goodfellow in 2014 [24]. Generally, a standard GAN comprises two modules, the generator and the discriminator. The generator learns the distribution of the training data, and a discriminator’s goal is to distinguish the samples of the original training set from the generated ones. This capability exhibited by the generative adversarial network has made its application in intelligent fault diagnosis. Pan et al. [25] reviewed the related literature on small sample-focused fault diagnosis methods using GANs. Their paper describes the GAN approaches and reviews GAN-based intelligent fault diagnosis applications in the literature while discussing the limitations and future road maps of GAN-based fault diagnosis applications. Li et al. [26] also presented research on GANs with a focus on the theoretical development and achievements of GANs while introducing and discussing the improved GAN methods and their variants.

Liu et al. [26] presented a rotating machinery fault diagnostics framework that is based on GANs and multisensor data fusion to generate synthetic data from the original data. Zhang et al. [27], Wang et al. [28], and Lv et al. [29] all made use of one-dimensional time-domain signals to generate synthetic data using GANs for classification and diagnosis of rotating machinery. Similarly, Li et al. [30], Wang et al. [31], Zheng et al. [32], and Wang et al. [33] used one-dimensional frequency domain signals, and Huang et al. [34] and Shi et al. [35] used two-dimensional images while Pan et al. [36], and Zhou et al. [37] used one-dimensional feature sets to generate synthetic data.

The original GAN has been extended into various forms, such as the Wasserstein GAN (WGAN), convolutional-based GANs, semi-supervised GANs, and condition-based GANs to enhance the quality of data synthesis and improve the training process. For instance, the complexities of controlling the adversarial process between generator and discriminator cause a mode collapse/gradient disappearance phenomenon leading to unsatisfactory data generation performance of the GAN models. To overcome this challenge, Arjovsky et al. [38] introduced the Wasserstein GAN to deal with the mode collapse phenomena. It provided a solution to the instability problem of GAN but had the challenge of weight clipping, which was addressed by Gao et al. [39] through the combination of WGAN with a gradient penalty. Zhang et al. [40] also attempted to solve the small sample problem, focusing on intelligent fault diagnosis via the multi-module gradient penalized GAN. The proposed method comprises three network modules: generator, discriminator, and classifier. The mechanical signals were generated by adversarial training and were then used as training data. References [41,42] also used GANs for the fault diagnosis problem of rotating machinery.

These improved variants of GANs have been extensively applied to roller-bearing fault diagnoses. There have also been many combinations of GANs with other generative models for fault diagnoses, namely encoder, autoencoder (AE), and variational autoencoder. Wang et al. [31] combined GAN and the conditional variational autoencoder to enhance the quality of generated samples for fault pattern recognition in planetary gearboxes. Reference [43] proposed an improved fault diagnosis approach to learn the deep features of the data by combining an encoder with GANs, integrating the discriminator with the deep regret analysis method to avoid the mode collapse by imposing the gradient penalty on it. Reference [43] also proposed a novel method called upgraded GAN, which is a combination of energy-based GANs, auxiliary-classifier, and conditional variational autoencoders. Some other applications of GANs for data argumentation in the literature for fault diagnoses were demonstrated by Liu et al. [44], who proposed a data synthesis approach using deep feature-enhanced GANs for roller bearing fault diagnoses; [45] used wavelength transform to extract image features from time-domain signals with GANs to generate more training samples and CNN for fault detection. Generative algorithms have proven to be beneficial for solving the small sample problem encountered when using data-driven approaches for intelligent fault diagnoses. This method is widely accepted and more improvements and modifications to the standard GAN have been embraced in the literature to develop highly effective models capable of detecting and classifying industrial fault data and other applications in intelligent fault diagnosis; it was also adopted in this research work to develop new fault samples.

## 3. Background

This section provides a brief introduction to the networks and algorithms that are used in the paper. The long short-term memory (LSTM) network is an important concept and is used for classification, and the CNN network is used in both the architecture of the data generation algorithm and the classifier. Conditional GAN is the base of N2FGAN. Understanding its architecture is essential for comprehending the image-to-image translation and N2FGAN.

### 3.1. LSTM

LSTM is a type of recurrent neural network (RNN) and is one of the most potent classifiers in machine learning. The network’s efficiency and impressive ability stem from the formulation of the network and its learning algorithms. The output of a network is influenced by the information from the previous and current inputs. RNNs are an extension of the feed-forward neural networks but are distinguished by their memory.

RNNs are dynamic systems [46] with an internal state at each time step of the classification resulting from the connections between higher layer neurons and the neurons in the lower layers as well as optional self-feedback connection(s). Initially developed RN networks, such as Elman and Jordan networks [47], had a limitation of looking back in time for more extended time steps due to the issue of vanishing or exploding gradients. Long short-term memory recurrent neural networks were developed to address this issue. LSTMs usually have three gates input, forget, and output gates, which learn overtime what information is essential; the input gates determine whether a piece of information is important and usually use simple sigmoid function units with activation ranges between 0 and 1 to control the signal into the gate. The forget gate helps to decide if a piece of information should be deleted or kept, while the output gate learns how to control access to cell content and helps to decide which information is worthy of impacting the output of the current time-step.

**Figure 2:**
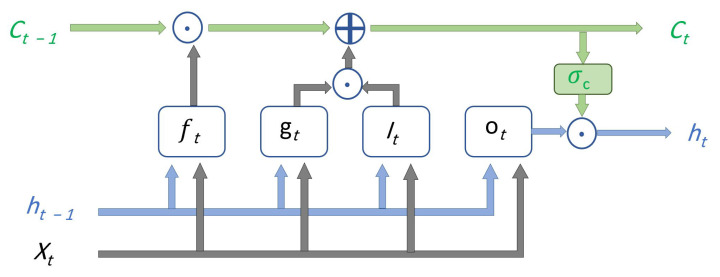
A basic structure of LSTM Network.

Figure 2 is the basic structure of LSTM-RNN, where $f_{t}$ is the forget gate, $g_{t}$ is the cell candidate, $i_{t}$ is the input gate, $o_{t}$ is the output gate, $C_{t}$ is the cell state, and $h_{t}$ is the hidden state.

### 3.2. CNN

Convolutional neural networks (CNNs) are artificial neural networks that are primarily used to solve image-driven pattern recognition tasks because of the design and structure of their architecture. They have been widely used in fault detection [48,49,50] because of their feature extraction capabilities. The idea behind convolution is to use kernels to extract particular features from input data.

**Figure 3:**
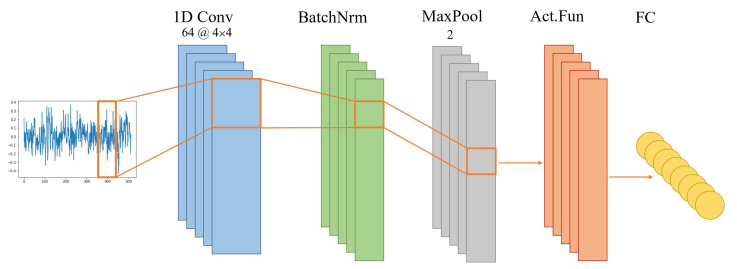
A schematic representation of the major CNN layers.

CNNs are composed of three main layers: the convolutional layer, pooling layer, and fully connected layer. The convolutional layer parameters use learnable kernels [51]. These kernels, usually two-dimensional for image recognition tasks or one-dimensional for time series data, glide over the entire depth of the input while calculating the scalar product for each kernel. Then an activation function is used to enhance the nonlinear expression of the convoluted features. The process is shown in Equation (1), where *x* is the signal, $f_{k}$ is the kernel filter, $b_{k}$ is the bias, and σ is the activation function. The pooling layers reduce the representation’s dimensionality, reducing the model’s computational complexity and allowing for better generalization. The most common pooling method in CNN is max-pooling max, which calculates the maximum value in a range *w*, as shown in Equation (2). The fully connected layer consists of neurons directly connected to the neurons in the two adjacent layers, similar to a traditional ANN. Figure 3 is a schematic representation of CNN with the convolution, batch normalization, pooling, activation function, and fully-connected layers [52].

(1) $$h_{k}=\sigma \left(x*f_{k}+b_{k}\right)$$

(2) $$h_{p}k=max\left(h_{k},w\right)$$

### 3.3. Conditional GAN

Generative adversarial networks consist of two networks trained simultaneously: (1) a generative model, *G*, capturing the data distribution to produce synthetic samples $\tilde{x}=G\left(z\right)$, while the input of the network is a random noise vector *z*, with the distribution of $P_{z}$; (2) a discriminator model, *D*, which discovers if the sample is generated by *G* or is a real sample from the training data. *G* is trained in a way to deceive *D* by making convincing, realistic samples. On the other hand, *D* estimates the corresponding probability of each sample to find out the source. The value function of GAN is defined in Equation (3).

(3) $$\begin{array}{c} \min_{G}\max_{D}V\left(D,G\right)=E_{x∼P_{r}}\left[\log\left(D\left(x\right)\right)\right]+E_{\tilde{x}∼P_{f}}\left[\log\left(1-D\left(\tilde{x}\right)\right)\right], \end{array}$$

where $P_{r}$ and $P_{f}$ denote the distribution of the raw data and the synthetic samples, respectively [24].

The conditional generative adversarial network is a variation of GAN. It places a condition on the generator and discriminator by feeding some extra information, *y*. This information could be data from other modalities. *y* is fed into both the generator and discriminator [15]. The objective of the CGAN is expressed in Equation (4).

(4) $$\begin{array}{c} \min_{G}\max_{D}V\left(D,G\right)=E_{x∼P_{r}}\left[\log\left(D\left(x|y\right)\right)\right]+E_{\tilde{x}∼P_{z}}[\log\left(1-D\left(G\left(z|y\right)\right)\right], \end{array}$$

### 3.4. Image-to-Image Translation

The proposed method used in this paper was inspired by the Pix2Pix, an image-to-image translation algorithm introduced by Phillip Isola et al. [18]. Image-to-image translation involves transforming an image from one domain to another, mapping an input image and an output image, or a day image to a night, for instance. Pix2Pix is the pseudonym for implementing a generic image-to-image translation solution that involves mapping pixels to pixels using CGANs. As mentioned earlier, GAN maps a random noise vector to an output, while CGAN learns mapping from an observation and random noise vector to the output. The framework used in [18] differs from other CGANs frameworks because it was designed not to be application-specific similar to image-to-image translation methods. The main characteristic of Pix2Pix compared to CGAN is that its generator’s input does not include random noise. This would make the output of the generator deterministic. To address this, the noise is added in the form of dropout layers to the generator’s architecture. Moreover, Pix2Pix chooses different architectures for its generator and discriminator, where U-Net, a convolutional network used for image segmentation, relies on data augmentation to use available samples more effectively [53], as well as PatchGAN [54], are used, respectively. Both use modules of the form convolution-BatchNorm-ReLU.

In our proposed work, a modification of Pix2Pix was adopted on vibration signals because of its generic nature and ability to work well on problems framed as an image-to-image translation.

## 4. Proposed Model (N2FGAN)

**Figure 4:**
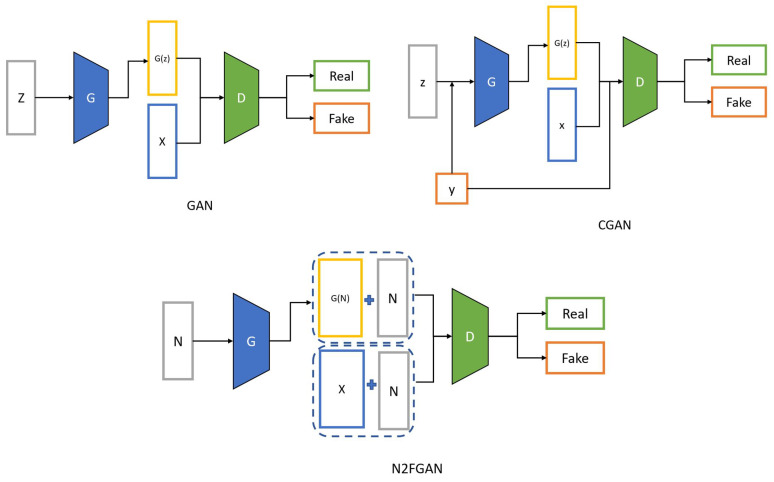
A comparison between GAN, CGAN, and our proposed methods(N2FGAN).

As mentioned before, fault data are scarce in industrial plants; however, normal data are ample. This section will introduce a novel method for generating fault data from normal data. This method is inspired by image-to-image translation, which is a variation of CGAN for the conditional data generation task. A conventional GAN learns mapping from the random noise vector *z* to the output synthetic sample $\tilde{x}$ = G(z). However, CGANs learn to map from observed information *y* and the random noise vector *z* to $\tilde{x}$. The CGAN model has been improved, making it suitable for signal-to-signal translation. In our proposed method (N2FGAN), similar to the Pix2Pix framework, normal data are used as the input of the generator without any random noise. In Figure 4, a comparison between GAN, CGAN, and N2FGAN is shown.

### 4.1. Network Architectures

#### 4.1.1. Generator

An encoder–decoder structure is used for the generator. The input is passed through some downsampling layers known as the encoder, and then the process is reversed in the decoder. In this process, hidden information of the data is extracted. The generator consists of an encoder and a decoder. Each block in the encoder consists of a 1D convolutional layer, a batch normalization layer, and a Leaky ReLU layer. Each block in the decoder consists of transposed convolution, batch normalization, dropout, and ReLU layers. There are skip connections between the encoder and decoder. The generator consists of four blocks in the encoder and decoder, where the input datum is a vector of length 512, and the dimension of the latent space is 64.

#### 4.1.2. Discriminator

The discriminator is a convolutional classifier. It includes three convolutional blocks, each consisting of a 1D convolutional layer, a batch normalization layer, and a leaky ReLU layer. It receives two pairs of concatenated inputs, the first inputs are the normal data and the actual fault data from the dataset, which should be classified as real data, and the second inputs are the concatenated normal data and generated fault data from the generator’s output, which is synthetic.

### 4.2. Objective

The total loss consists of the generator and the discriminator losses. The generator loss is a sigmoid cross-entropy loss of the generated data and an array of ones. To make generated data structurally similar to the target data, the L1 distance was used. The L1 loss is defined as the mean absolute error between the generated data and the target data.

(5) $$\begin{array}{c} L_{L1}\left(G\right)=E_{x,\tilde{x},z}\left[|\right|\tilde{x}-{G\left(x,z\right)||}_{1}] \end{array}$$

The discriminator loss consists of the sum of the sigmoid cross-entropy loss of the actual data and array of ones and the sigmoid cross-entropy loss of the generated data and array of zeros, respectively. The final objective of the proposed method is defined as follows, where λ is a constant and is considered 100.

(6) $$\begin{array}{c} G^{*}=\min_{G}\max_{D}V\left(D,G\right)+\lambda *L_{L1}\left(G\right), \end{array}$$

## 5. Experiments and Discussion

**Figure 5:**
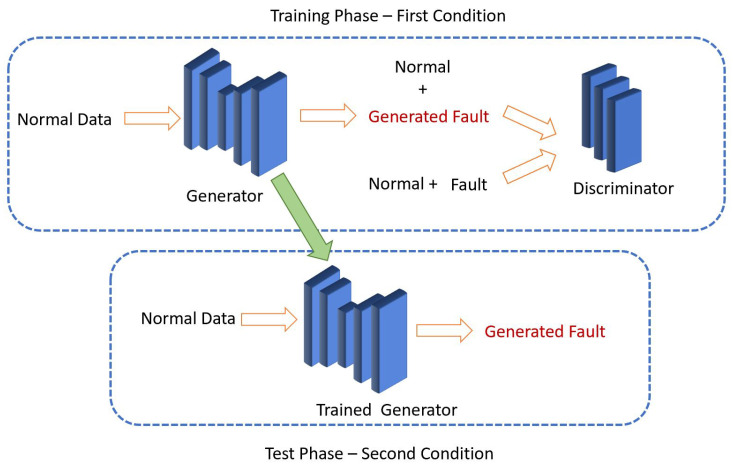
The proposed data generation framework (N2FGAN).

In the following, N2FGAN is implemented for generating new fault data. The network is trained in the first condition, where both normal and fault data are available. In the next step, the trained generator makes fault data from normal in a new condition where there is no fault data available. The model is tested on a real-world dataset in various conditions. Finally, three classifiers are used for the evaluation of the generated data. In this case, the machinery motor loads and motor speeds are considered different conditions. Some statistical features of the generated data are compared with the actual data, and some visualizations are also created to show the quality of the generated data. In this paper, actual data are referred to as the samples from the dataset, and generated data are the output of the generator network. An overview and steps of the proposed method are shown in Figure 5. All experiments were performed using Python 3.7 on a computer with a GPU of NVIDIA Tesla P100 and 16 GB of memory.

### 5.1. Dataset Description

**Figure 6:**
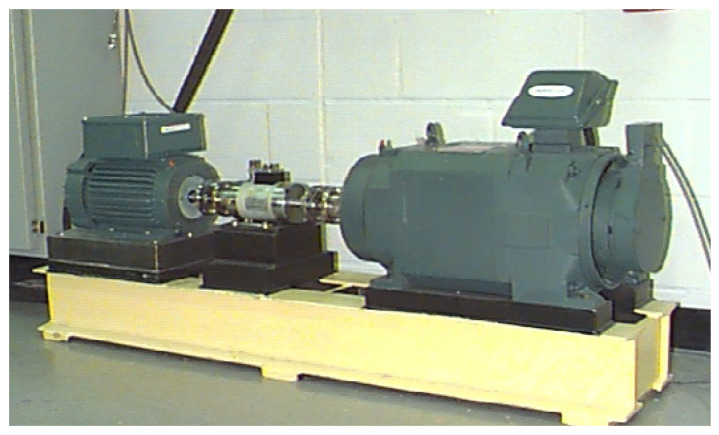
CWRU bearing data collection test bed.

In this paper, a CWRU-bearing dataset (https://engineering.case.edu/bearingdatacenter (accessed on 20 June 2022)) was used for data generation. The testbed is shown in Figure 6. The data were collected using an experimental setup consisting of a 2 hp Reliance Electric motor, a torque transducer/encoder, a dynamometer, and control electronics. Acceleration data were collected at 12,000 samples/second from the fan-end and drive-end of the machine. Faults were made artificially using electro-discharge machining (EDM), and the diameter of faults ranged from 0.007 inches to 0.040 inches. There were three fault categories, inner raceway, rolling element, and outer raceway. The outer race fault was collected in three different orientations directly in the load zone, orthogonal to the load zone, and opposite to the load zone, so there were five different types of faults in total. Vibration data were recorded for motor loads between 0 and 3 horsepower with speeds of 1730 to 1797 RPM. In the experiment, drive-end data were used. To generate different datasets, a signal burst of length 200 was used, and 100 SNR noise was added to make data generation more complex.

### 5.2. Training Phase of the Data Generation Algorithm

**Figure 7:**
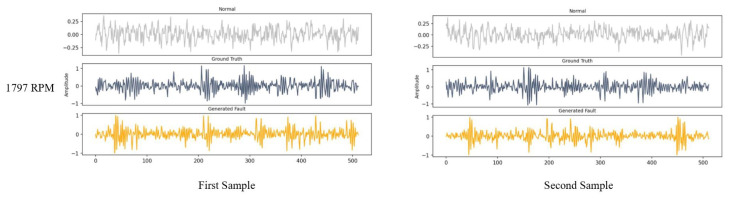
Some samples of generated fault data in 1797 RPM.

The defined architecture was used to generate fault data in the first condition (in this case first RPM), where both normal and fault data were available. The process was done by applying the Adam optimizer with a learning rate of 0.0002 over 4000 steps. The experiment was conducted on actual normal and inner race faults with a diameter of 0.007 inches at 1797 RPM. In this case, although the fault data were available, more fault data were generated. Some samples of the normal and actual fault data, also known as ground truth and generated fault data in the training phase, are shown in Figure 7.

### 5.3. Data Generation in New Condition

**Figure 8:**
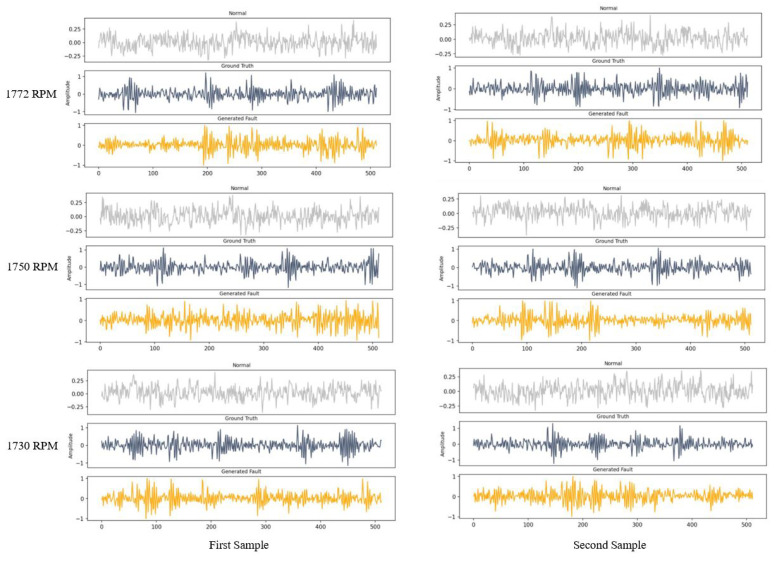
Some samples of the generated fault data for different RPMs.

The trained generator was used for generating fault data in new conditions; these conditions are defined as different working speeds at 1772, 1750, and 1730 RPM, where it has been assumed that there is no sample of fault data; however, the generated fault data were compared to the actual data for evaluation. The generator’s input was normal data in new conditions, and the fault data were not used in the data generation process. Some samples of the generated data in the different conditions using the trained network are shown in Figure 8. The generator was trained in 1797 RPM, and the same network was used for data generation in all conditions.

### 5.4. Evaluation

**Figure 9:**
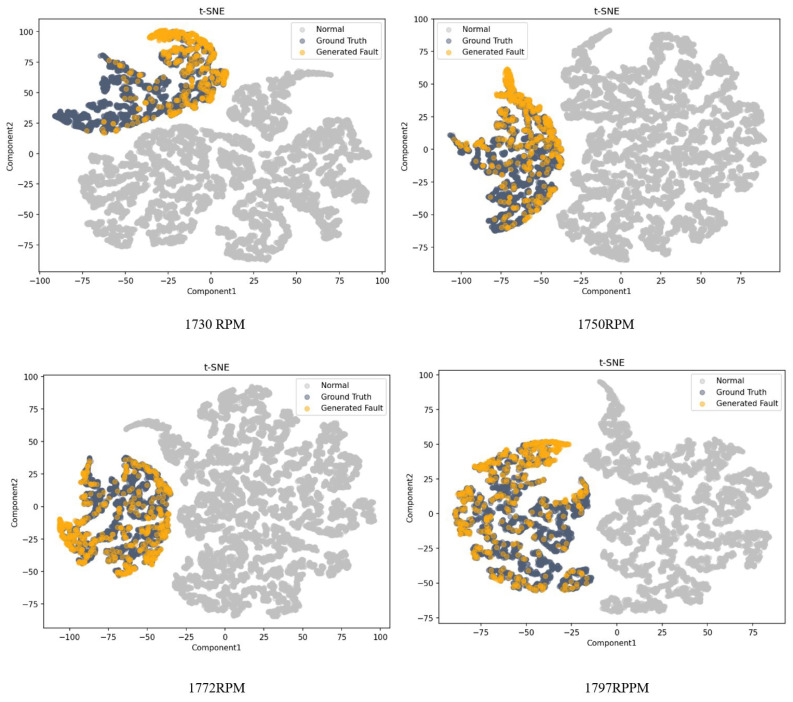
t-SNE visualization of the generated data.

Evaluating the quality of the generated data is a difficult task. The generated samples should be similar to the actual data in any conditions. Neural network-based classifiers were used to validate the generated data alongside a statistical comparison with the t-distributed stochastic neighbor embedding (t-SNE) to represent the statistical distributions. Both time domain and frequency domain features were extracted based on work in [55] and used for the t-SNE visualization. The selected features are listed in Table 1 and the two-component t-SNE plots for different conditions are shown in Figure 9. The generated fault data are easily separable from normal condition data and are very similar to the ground truth, which shows that the distributions of the generated signal features closely match the distributions of actual fault features.

In the first step, a binary LSTM classifier with a Softmax layer was used to determine if the generated data were faulty or normal [56]. The classifier was trained on the actual dataset. After the training phase, the actual data of the target class were replaced with the generated data samples and fed into the classifier as test data. The test data were composed of normal, inner race, ball, the outer race centered, outer race orthogonal, and outer race opposite fault data with 0 to 5 labels, respectively; there were 480 samples in each class. The experiment was conducted several times on binary and multiclass classifiers. The binary classifier’s accuracy for the generated data in the same condition and the new condition was 100%, which showed that the generated fault data were not similar to normal. Three multiclass classifiers, convolutional LSTM (ConvLSTM), CNN, and convolutional autoencoder (ConvAE), were used in this experiment to evaluate the effectiveness of adding synthetic samples. Their details are shown in Table 2. The multi-class classifiers also illustrate a high performance, having more than 97% accuracy. Table 3 shows the performance of the classifiers applied to the generated data.

In Table 4, different N2FGAN architectures are compared, the first condition is 1797 RPM and the second condition is 1772 RPM. All experiments were conducted by considering 40,000 steps for training the network. Three different architectures were considered for the generator (G) with 3, 4, and 5 blocks and the discriminator(D) with 2, 3, and 4 blocks. The number of neurons is mentioned in the table. These blocks were connected to make the networks. The results show that the deep networks perform better and the training time is longer. Different input data lengths of 256,512 and 1024 samples were also studied. According to the table, N2FGAN can generate more accurate data, while the input data size is large enough and performs better with lengths of 512 and 1024. No significant difference in performance was found between these two last lengths.

In order to evaluate the performance of N2FGAN compared to other similar algorithms, a comparison panel, including classical augmentation and two state-of-the-art generative algorithms, Wasserstein GAN with gradient penalty (WGAN-GP) and CGAN, were chosen. Classical augmentation is a set of operations, such as reversing the signal burst, adding Gaussian noise to it, and flipping it by multiplying its values to minus one [56]. In this experiment, ConvLSTM was trained on all six classes. As for the health class, it was trained on both motor speeds of 1797 and 1772 RPM. There were 250 training samples for the inner class, including 150 real samples for the motor speed of 1797 and 100 samples generated using different augmentation frameworks in the comparison panel. As discussed earlier, N2FGAN takes the first condition (1797 RPM) and generates the second condition (1772 RPM), while the rest of the frameworks can only augment the first condition. As for the other four classes, only 150 real samples in the first condition were used to train the classifier. Table 5 exhibits the number of real and synthetic samples as well as the training conditions and the test conditions for different CWRU classes. In this experiment, the number of training samples for all the fault classes was set relatively low to resemble real-world situations where the practitioners have to deal with imbalanced and insufficient data.

**Figure 10:**
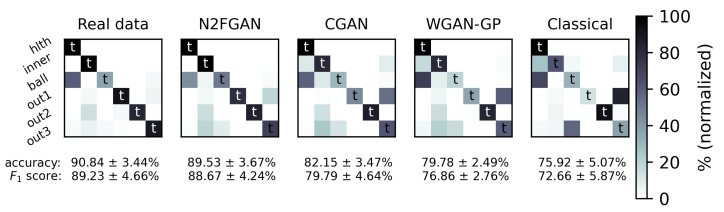
Comparing the effect of each augmentation framework on the classifier performance when the inner class is augmented; *t* stands for true labeled classes.

The experiment was run 20 times, each time with different training and test sets collected from the CWRU dataset. As can clearly be seen in Figure 10, N2FGAN outperforms the other augmentation frameworks. In fact, the results demonstrate that the performances of the classifiers trained on the real data and the data generated by N2FGAN were very similar. Classical augmentation had rather poor effectiveness since the average accuracy of the classifier trained on a non-augmented dataset was about 73.2%, only 2% less than classical augmentation. CGAN and WGAN-GP had mediocre effectiveness as they had only improved the accuracy by almost 9% and 6%, respectively.

## 6. Conclusions

In industrial environments, fault data are scarce, and in many cases, normal data are abundant. Machines work in different conditions (i.e., numerous motor loads and speeds), for which fault samples are rarely available. This makes the utility of any machine learning-based method limited since the developed model will be greatly biased to normal conditions. By augmenting normal data with sufficient fault data in a certain condition, the proposed framework enables machine learning-based models that are more robust for fault diagnoses, even in unforeseen fault conditions.

This paper introduces a novel data augmentation algorithm to synthesize fault data. In this algorithm, a variation of CGAN is proposed that can be trained on normal and fault data of one condition. The trained generator of the network was used to generate fault data from the normal samples for each motor speed for which there were no fault data available. The generated data were compared with the actual data and the normal input data using t-SNE. The results illustrate that the generated fault data have the same characteristics as the real fault data.

Moreover, three different classifiers were employed to validate the quality of the synthesized data. The classifiers were trained on various normal and actual fault samples. For the test phase, a new dataset was extracted from the primary dataset with the actual faults from the target class replaced by generated faults and fed into the trained classifiers for testing. In our experiments, three different conditions were tested with respect to different motor speeds. The results demonstrate that the generated faults are correctly classified with high accuracy (more than 97% in all cases). This proves that the generated fault data are very similar to the actual fault data. On the other hand, three frameworks were provided (including CGAN and WGAN) to evaluate the effectiveness of the proposed model in an imbalanced condition. N2FGAN, compared to the others, has demonstrated a higher similarity to the real data and improved the classification performance significantly.

Future extensions of the present work will focus on exploring the effectiveness of generating the signal features instead of the raw vibration samples. In addition, the work should explore an efficient hyperparameter tuning framework to train the generator faster without compromising its performance. Furthermore, reducing the complexity of the network to reduce the training time can be another avenue for future work.

## Figures and Tables

**Table 1:** Selected features for analysis of the generated fault data.

Time Domain Feature	Formula	Frequency Domain Feature	Formula
Mean	$\bar{x}=\frac{1}{N}\sum_{i=1}^{N}x\left(i\right)$	Mean	$\bar{f}=\frac{1}{N}\sum_{i=1}^{N}f\left(i\right)$
Standard Deviation	$\sigma =\sqrt{\frac{1}{N-1}\sum_{i=1}^{N}\left(x\left(i\right)-\bar{x}\right)}$	Standard Deviation	$\sigma_{f}=\sqrt{\frac{1}{N-1}\sum_{i=1}^{N}\left(f\left(i\right)-\bar{f}\right)}$
Skewness	${\tilde{\mu }}_{3}=\frac{\sum_{i=1}^{N}{\left(x\left(i\right)-\bar{x}\right)}^{3}}{\left(N-1\right)*\sigma^{3}}$	Skewness	${\tilde{\mu }}_{3f}=\frac{\sum_{i=1}^{N}{\left(x\left(i\right)-\bar{f}\right)}^{3}}{\left(N-1\right)*\sigma_{f}^{3}}$
Crest Factor	$CF=\frac{max|x(i)|}{\sqrt{\frac{1}{N}\sum_{i=1}^{N}x{\left(i\right)}^{2}}}$	Crest Factor	$CF_{f}=\frac{max|f(i)|}{\sqrt{\frac{1}{N}\sum_{i=1}^{N}f{\left(i\right)}^{2}}}$
Kurtosis	$\kappa =\frac{1}{N}\sum_{i=1}^{N}x\left(i\right)-max{\left(x\left(i\right)\right)}^{4}$	Shannon Entropy	$-\sum_{i=1}^{N}f(i)log(f(i))$

**Table 2:** Classifier descriptions.

Framework	Description
ConvLSTM	The architecture consists of two CNN blocks (containing 1D-convolutional layers, batch normalization, ReLU, and max pooling,), an LSTM block, a dense layer with sigmoid activation function, a dropout, and a SoftMax layer.
CNN	It consists of four CNN blocks (containing one 1D-convolutional layer, Batch Normalization, ReLU, and Max Pooling layer), A flattened layer, a fully connected layer, and a SoftMax classification layer.
ConvAE	It is a multi-layer network consisting of an encoder and a decoder. Each includes three CNN blocks (containing 1D-convolutional layers, ReLU, and max pooling, or upsampling),a flattened, a fully connected layer, and a SoftMax classification layer.

**Table 3:** Classifier accuracy, $F_{1}$ score, precision, and recall for test data in different conditions while the training condition is 1797 RPM.

Condition	ConvLSTM Classifier				CNN Classifier				ConvAE Classifier			
	**Accuracy**	$F_{1}$ **Score**	**Precision**	**Recall**	**Accuracy**	$F_{1}$ **Score**	**Precision**	**Recall**	**Accuracy**	$F_{1}$ **Score**	**Precision**	**Recall**
1797	98.89%	98.89%	98.9%	98.89%	99.34%	99.34%	99.35%	99.34%	99.38%	99.37%	99.38%	99.37%
1772	98.78%	98.78%	98.81%	98.78%	98.85%	98.85%	98.89%	98.85%	99.27%	99.70%	99.28%	99.27%
1750	99.24%	99.24%	99.24%	99.24%	98.47%	98.47%	98.6%	98.47%	98.65%	98.65%	98.66%	98.65%
1730	98.72%	98.71%	98.74%	98.72%	98.61%	98.61%	98.63%	98.61%	97.57%	97.57%	97.65%	97.57%

**Table 4:** Comparison between different architectures of the N2FGAN tested for 1772 RPM.

				ConvLSTM Classifier				CNN Classifier				ConvAE Classifier			
**Runtime (s)**	**Generator Blocks**	**Discriminator Blocks**	**Input Size**	**Accuracy**	$F_{1}$ **Score**	**Precision**	**Recall**	**Accuracy**	$F_{1}$ **Score**	**Precision**	**Recall**	**Accuracy**	$F_{1}$ **Score**	**Precision**	**Recall**
535.71	3(Input length-256-64)	2(64-256)	256	90.94%	90.37%	91.81%	90.94%	92.57%	92.45%	92.92%	92.6%	92.50%	92.48%	92.79%	92.50%
682.18	3(Input length-256-64)	2(64-256)	512	98.54%	98.53%	98.60%	98.40%	97.67%	97.66%	97.75%	97.67%	91.87%	91.57%	93.79%	91.87%
1282.18	3(Input length-256-64)	2(64-256)	1024	99.2%	99.20%	99.23%	99.20%	99.72%	99.72%	99.73%	99.72%	99.34%	99.34%	99.36%	99.34%
674.56	4(Input length-256-128-64)	3(64-128-256)	256	94.44%	94.35%	94.81%	94.44%	92.20%	92.01%	93.00%	92.19%	88.89%	88.74%	90.16%	88.89%
1346.31	4(Input length-256-128-64)	3(64-128-256)	512	98.78%	98.78%	98.81%	98.78%	98.85%	98.85%	98.89%	98.85%	99.27%	99.70%	99.28%	99.27%
1381.87	4(Input length-256-128-64)	3(64-128-256)	1024	98.10%	98.05%	98.21%	98.06%	99.83%	99.83%	99.83%	99.83%	86.60%	84.12%	92.16%	86.60%
775.10	5(Input length-512-256-128-64)	4(64-128-256-512)	256	81.11%	74.74%	71.00%	81.11%	81.11%	74.96%	71.36%	81.11%	78.37%	72.21%	69.11%	78.37%
1102.08	5(Input length-512-256-128-64)	4(64-128-256-512)	512	99.24%	99.23%	99.25%	99.24%	98.26%	98.26%	98.35%	98.26%	96.15%	96.11%	96.74%	96.15%
1812.25	5(Input length-512-256-128-64)	4(64-128-256-512)	1024	99.72%	99.72%	99.72%	99.72%	98.04%	98.38%	98.48%	98.4%	88.02%	86.10%	92.20%	88.02%

**Table 5:** Training and test set configuration for comparing N2FGAN, CGAN, WGAN, and classical augmentation.

Classes	Training Set			Test Set	
	RPM	#Real Samples	#Synthetic Samples	RPM	#Real Samples
health	1797 and 1772	3000	0	1772	150
inner	1797	150	100	1772	150
ball	1797	150	0	1772	150
outer1	1797	150	0	1772	150
outer2	1797	150	0	1772	150
outer3	1797	150	0	1772	150

## Data Availability

https://engineering.case.edu/bearingdatacenter (accessed on 20 June 2022).

## Nomenclature

The following abbreviations are used in this manuscript:

**Abbreviations**	
AE	Autoencoder
AI	Artificial Intelligence
CGAN	Conditional Generative Adversarial Networks
CNN	Convolutional Neural Network
ConvAE	Convolutional Auto Encoder
ConvLSTM	Convolutional LSTM
CWRU	Case Western Reserve University
DBN	Deep belief network
EDM	Electro-discharge machining
GAN	Generative adversarial network
IFD	Intelligent fault diagnosis
k-NN	K-nearest neighbor
LSTM	Long short-term memory
N2FGAN	Normal to fault GAN
RNN	Recurrent neural network
RPM	Revolutions per minute
SVM	Support vector machine
t-SNE	t-Distributed stochastic neighbor embedding
VAE	Variational autoencoder
WGAN	Wasserstein GAN
**Symbols**	
$c_{n}$	Working conditions
$f_{k}$	Kernel filter
$b_{k}$	Bias
σ	Activation function
$P_{r}$	Distribution of the raw data
$P_{f}$	Distribution of the the fake samples
*y*	Extra information
*z*	Random noise vector
*G*	Generative model
*D*	Discriminator model
$f_{t}$	Forget gate
$g_{t}$	Cell candidate
$i_{t}$	Input gate
$o_{t}$	Output gate
$C_{t}$	Cell state
$h_{t}$	Hidden state
